# Crosstalk between tongue carcinoma cells, extracellular vesicles, and immune cells in *in vitro* and *in vivo* models

**DOI:** 10.18632/oncotarget.17768

**Published:** 2017-05-10

**Authors:** Ahmed Al-Samadi, Shady Adnan Awad, Katja Tuomainen, Yue Zhao, Abdelhakim Salem, Mataleena Parikka, Tuula Salo

**Affiliations:** ^1^ Department of Oral and Maxillofacial Diseases, Clinicum, University of Helsinki, Helsinki, Finland; ^2^ Hematology Research Unit, Department of Hematology, University of Helsinki and Helsinki University Central Hospital Comprehensive Cancer Center, Helsinki, Finland; ^3^ Clinical Pathology Department, National Cancer Institute, Cairo University, Cairo, Egypt; ^4^ Department of Otorhinolaryngology, Helsinki University Hospital, Helsinki, Finland; ^5^ Department of Internal Medicine, Clinicum, University of Helsinki, Helsinki, Finland; ^6^ Faculty of Medicine and Life Sciences, University of Tampere, Tampere, Finland; ^7^ Oral and Maxillofacial Unit, Tampere University Hospital, Tampere, Finland; ^8^ Cancer and Translational Medicine Research Unit, University of Oulu, Oulu, Finland; ^9^ Medical Research Center, Oulu University Hospital, Oulu, Finland

**Keywords:** tongue cancer, immune cells, extracellular vesicles, *in vitro* model, cytotoxicity

## Abstract

The crosstalk between immune cells, cancer cells, and extracellular vesicles (EVs) secreted by cancer cells remains poorly understood. We created three-dimensional (3D) cell culture models using human leiomyoma discs and Myogel to study the effects of immune cells on highly (HSC-3) and less (SCC-25) invasive oral tongue squamous cell carcinoma (OTSCC) cell lines. Additionally, we studied the effects of EVs isolated from these cell lines on the cytotoxicity of CD8^+^ T and NK cells isolated from three healthy donors. Our analysis included the effects of these EVs on innate immunity in zebrafish larvae. Activated immune cells significantly decreased the proliferation of both OTSCC cell lines and associated with a diminished invasion area of HSC-3 cells. In general, EVs from SCC-25 increased the cytotoxic activity of CD8^+^ T and NK cells more than those from HSC-3 cells. However, this effect varied depending on the source and the immune and cancer cell subgroups. In zebrafish, the amount of IL-13 mRNA was decreased by SCC-25 EVs. This study describes promising *in vitro* and *in vivo* models to investigate interactions between immune cells, cancer cells, and EVs.

## INTRODUCTION

After the introduction of Coley's toxin, “a suspension of the gram-negative bacteria,” as an activator of the immune system against cancer cells, researchers turned their attention to the role of the immune system in cancer research [1]. Subsequently, studies showed that the immune response to cancer may either suppress or support tumor growth depending on the type of immune effector mechanism activated. Based on these studies, immune cells can be broadly divided into “good/anti-tumorigenic immune cells” represented by, for instance, Th1, CD8^+^T, NK, and M1 macrophages and “bad/pro-tumorigenic immune cells” such as Th2, Treg, and M2 macrophages [2].

In oral tongue squamous cell carcinoma (OTSCC), the most common type of head and neck cancer, a lymphocytic infiltrate was associated with a better response to radiotherapy and an overall good prognosis [3]. More specifically, our group has already found a correlation between inflammatory cell infiltrates and OTSCC prognosis depending on these cell types [4]. That is, we found that patients with a tumor microenvironment (TME) rich in CD163^+^Foxp3^+^ CD80^+^ experience a higher rate of cancer recurrence compared to patients with TME low in CD163^+^Foxp3^+^ CD80^+^ [4].

Extracellular vesicles (EVs) or exosomes are small vesicles (30–100 nm) released from all cells [5] which carry various proteins, lipids, and nucleic acids (DNA, mRNA, and miRNA) and are found in biological fluids including saliva, blood, and cell culture media [6]. Interestingly, tumor cells were found to secrete more EVs than normal cells [7]. Thus, it became clear that cancer cells use EVs as a tool for distant communication with other cells, including TME, through the horizontal transfer of their active biomolecules. In fact, EVs seem to play an active role in the biology and clinical course of cancer by modulating the immune system and affecting the cell phenotype [8–10].

This study aimed to identify better *in vitro* TME matrix 3D models for co-culturing immune and cancer cells and to investigate the crosstalk between these cells. First, we discuss the effects of immune cells on the proliferation, migration, and invasion of OTSCC cells *in vitro* using human myoma discs and a soluble myoma matrix “Myogel” in 3D cell culture models. Then, we describe our analysis of the effects of EVs from OTSCC cells on the phenotype and cytotoxic activity of selected immune cells *in vitro* and on the innate immune system *in vivo* using a zebrafish model.

## RESULTS

### Association between activated peripheral blood mononuclear cells and OTSCC cell proliferation and invasion area in myoma discs

**Figure 1:**
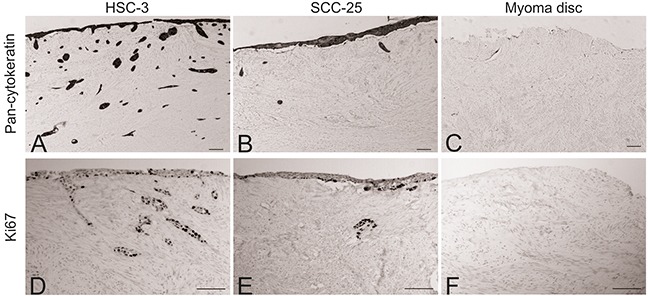
Comparison of the invasion ability of HSC-3 SCC-25 cells Myoma discs (with or without OTSCC cells) were stained with pan-cytokeratin and Ki67. HSC-3 showed a higher ability to invade compared to SCC-25 **(A** and **B)**, while no staining was found in the myoma discs without cancer cells **(C)**. The percentage of Ki67^+^ cells was similar for HSC-3 and SCC-25 cells **(D** and **E)**; similar to pan-cytokeratin, the myoma discs without cancer cells were negative for Ki67 **(F)**. Scale bar = 100 μm.

**Figure 2:**
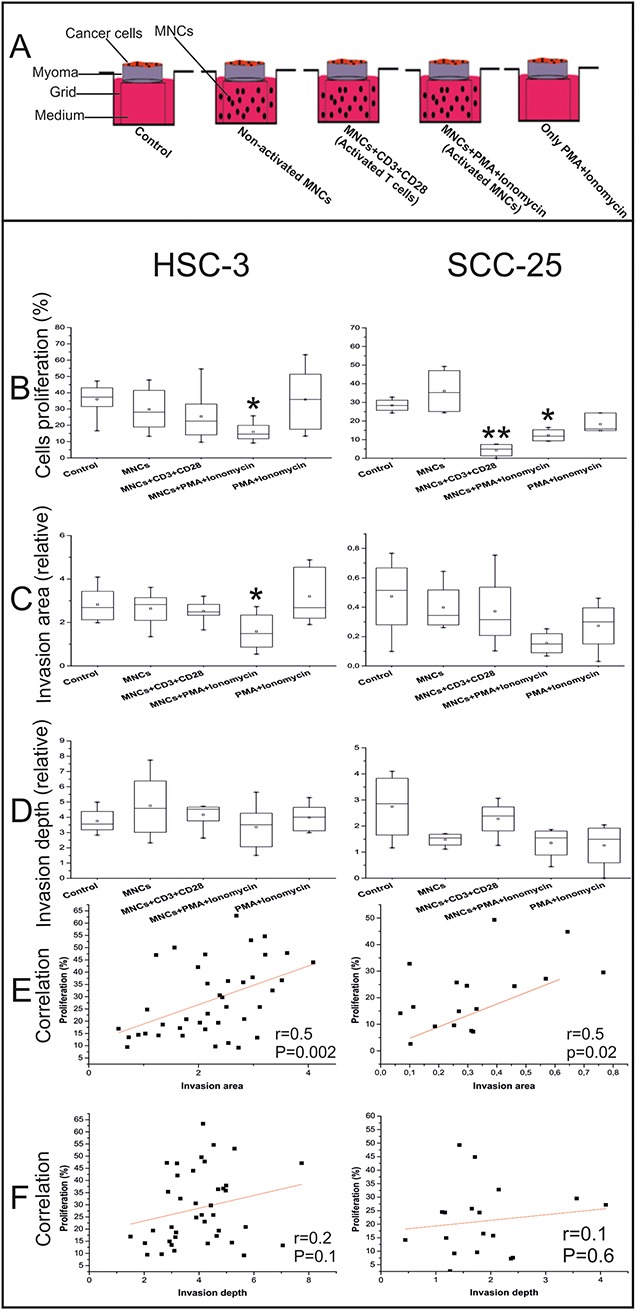
Effects of the peripheral blood MNCs on the OTSCC cell proliferation, invasion area, and invasion depth Schematic of our co-culture model of cancer and immune cells **(A)**. The percentage of Ki67^+^ cells were counted from at least three randomly selected fields **(B)**. Invasion area **(C)** and invasion depth **(D)** were measured using the ImageJ software. Correlations between cell proliferation and invasion area **(E)** and invasion depth **(F)** were completed using the Pearson's test. We used the one-way ANOVA followed by Tukey's post-hoc test to determine statistical significance. *P ≤ 0.05, **P ≤ 0.01. All experiments were repeated independently four to eight times and each in duplicate.

After co-culturing the peripheral blood mono-nuclear cells (MNCs) with OTSCC cells in a 3D organotypic model, myoma discs were prepared for immunohistochemical staining for pan-cytokeratin and Ki67. In accordance with previous reports, HSC-3 cells showed a higher invasion ability compared with SCC-25 cells (Figure 1A and 1B). No positive staining for pan-cytokeratin was detected in myoma discs without cancer cells (Figure 1C). The percentage of Ki67^+^ cells was similar for HSC-3 and SCC-25 cells on the surface of the myoma, that is, cells had not invaded the discs (Figure 1D and 1E). Similar to pan-cytokeratin, myoma discs without cancer cells were negative for Ki67 (Figure 1F). Figure 2A illustrates our co-culture model of the OTSCC cells and MNCs.

We found that activated MNCs reduced cancer cell proliferation (both HSC-3 and SCC-25, *P*=0.04 and *P*=0.03, respectively). Yet, when CD3^+^ T cells were activated, the reduction effect was only found for SCC-25 cells (*P*=0.002; Figure 2B).

In line with a decrease in cancer cell proliferation, the invasion area in the myoma discs was reduced when activated MNCs were added. This reduction was statistically significant for the HSC-3 cells (*P*=0.04), but not for SCC-25 cells, for which the invasion area was much less than for HSC-3 cells (Figure 2C).

In addition, the invasion depth for both HSC-3 and SCC-25 cells was not affected by adding the peripheral blood MNCs (Figure 2D).

In both tongue cancer cell lines, cell proliferation positively correlated with the invasion area (r=0.5, *P*=0.002 for HSC-3; r=0.5, *P*=0.02 for SCC-25; Figure 2E), but not with invasion depth (r=0.2, *P*=0.1 for HSC-3; r=0.1, *P=*0.6 for SCC-25; Figure 2F).

### Migration of peripheral blood MNCs toward OTSCC cells through myoma discs

To study the migration of peripheral blood MNCs toward OTSCC cells through the myoma discs, we stained myoma slides with CD45 (a common leukocyte antigen) and CD8 (a cytotoxic T cell marker). Because myoma tissue already has its own inflammatory cells, CD45^+^ and CD8^+^ T cells were counted and compared between different groups. All myoma discs used in this study originated from a single batch.

**Figure 3:**
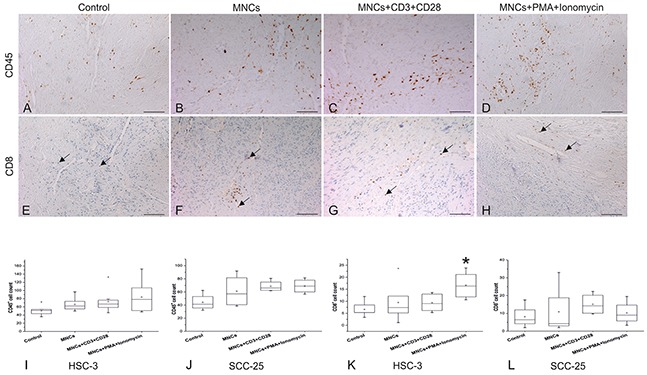
Migration of peripheral blood MNCs toward OTSCC cells through myoma discs Slides from the myoma discs were stained with CD45 **(A-D)** and CD8 **(E-H)**, images presented are from our HSC-3 experiment. The number of CD45^+^ **(I, J)** and CD8^+^ T cells **(K, L)** were calculated from at least three selected fields (10 or 20X magnification) showing the highest infiltration for each slide. We used the one-way ANOVA followed by Tukey's post-hoc test to determine statistical significance. *P ≤ 0.05. All experiments were repeated independently four to eight times and each in duplicate. Scale bar = 100 μm.

We found that CD45^+^ cells became more numerous in the myoma discs when activated MNCs were added (Figure 3A–3D). We found a similar trend for CD8^+^ T cells, primarily at the bottom of the myoma discs around the blood vessels (Figure 3E–3H).

In addition, CD45^+^ cells increased in the activated MNCs group across nearly all experiments; however, this finding was not statistically significant due to fluctuations in the number of CD45^+^ cells from one sample to another (Figure 3I and 3J).

Yet, the increase in CD8^+^ T cells was statistically significant in the presence of highly invasive HSC-3 cells (*P*=0.02), but not for less invasive SCC-25 cells (*P*>0.05; Figure 3K and 3L).

Adding MNCs without CD3 and CD28 antibodies or phorbol 12-myristate 13-acetate (PMA) and ionomycin appeared insufficient to prompt the migration of peripheral blood MNCs toward OTSCC cells (Figure 3I–3L).

### The effect of peripheral blood MNCs on the migration of OTSCC cells on Myogel

We used a co-culture of the peripheral blood MNCs with HSC-3 and SCC-25 cells in 96-well plates coated with Myogel to test whether the presence of peripheral blood MNCs affects the migration of cancer cells (Supplementary Video 1). We found that peripheral blood MNCs exerted no effect on the migration of HSC-3 or SCC-25. Primary inhibition was achieved by adding MNCs with PMA and ionomycin. However, PMA and ionomycin alone also appeared to inhibit migration (Supplementary Figure 1).

### The effect of EVs from OTSCC cells on human primary macrophage polarization

We differentiated human primary monocytes to macrophages for seven days using a macrophage-colony stimulating factor (M-CSF; Supplementary Figure 2A).

Macrophages were incubated for 24 hours with the HSC-3 and SCC-25 EVs and polarized to M1 using lipopolysaccharide (LPS) and interferon gamma (IFN-γ) and M2 using interleukin (IL)-4 and IL-13 as the positive controls. M1 macrophages were rounded, while M2 macrophages were more cone-shaped. We found that macrophages incubated with HSC-3 and SCC-25 EVs were primarily similar to spindle-shaped M0 with some cells exhibiting the M2-representative cone-shaped morphology (Supplementary Figure 2B). To study the effect of the OTSCC cell EVs on macrophage polarization, we used quantitative real-time PCR for the M1 (CXCL9 and CXCL10) and M2 (CCL17, CCL18, and CCL22) markers.

Macrophages incubated with HSC-3 and SCC-25 EVs exhibited no difference from controls for any of the M1 and M2 markers (Supplementary Figure 2C). As a positive control, a macrophage stimulated with LPS and IFN-γ strongly expressed the M1 markers (CXCL9 and CXCL10), while the macrophages stimulated with IL-4 and IL-13 highly expressed the M2 markers (CCL17, CCL18, and CCL22; Supplementary Figure 2C).

### EVs from OTSSC cells modulate the cytotoxic activity of CD8^+^ T and NK cells

We isolated human primary CD8^+^ T and NK cells from three healthy donors and incubated them with HSC-3 or SCC-25 EVs for 24 hours. Then, we added CD8^+^ T and NK cells to HSC-3 and SCC-25 cells to study their cytotoxic activity (Supplementary Video 2 and 3).

**Figure 4:**
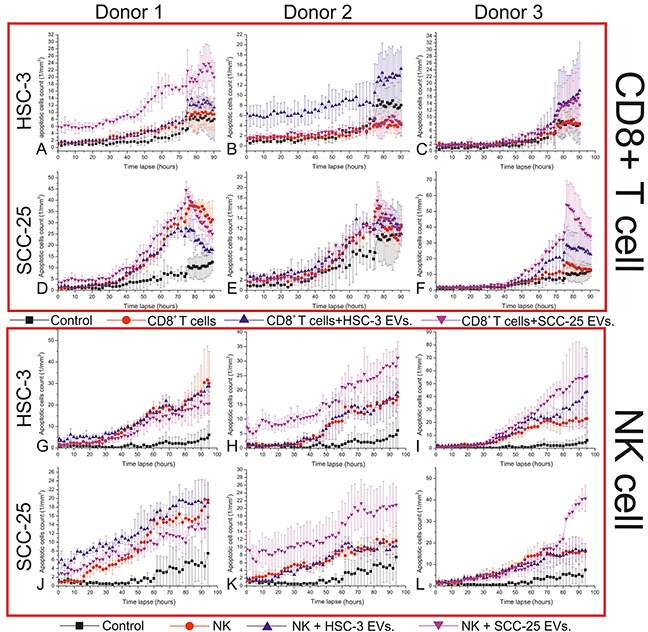
The effects of the EVs from OTSCC cells on the cytotoxic activity of CD8+ T and NK cells An immune killing assay was used to study the effect of OTSCC cell EVs on the cytotoxic activity of CD8^+^ T and NK cells. CD8^+^ T and CD56^+^ NK cells were isolated from the buffy coat of three healthy donors and incubated with media alone, HSC-3 EVs or SCC-25 EVs, for 24 hours. CD8^+^ T and NK cells were then incubated with HSC-3 and SCC-25 cells for five days and the number of apoptotic cells were counted over time.

In general, HSC-3 and SCC-25 EVs increased the cytotoxic activity of CD8^+^ T and NK cells (Figure 4). However, this effect varied, depending upon several factors including the following: the source of the immune cells (cells from different donors showed different responses to cancer cell exosomes), the source of EVs (from HSC-3 or SCC-25 cells), the type of affected cancer cells (HSC-3 or SCC-25 cells), and the type of immune cells (CD8^+^ T or NK cells). The cytotoxic activity of the CD8^+^ T cells from donor 1 increased due to SCC-25 EVs when added to HSC-3 but not SCC-25 cells (Figure 4A and 4D). For donor 2, HSC-3 EVs appeared most effective, but only against HSC-3 (Figure 4B and 4E). For donor 3, both HSC-3 and SCC-25 EVs increased the cytotoxic activity of CD8^+^ T cells against both HSC-3 and SCC-25 cells (Figure 4C and 4F).

For NK cells, HSC-3 and SCC-25 EVs did not induce the cytotoxic activity of NK cells from donor 1 (Figure 4G and 4J). For donor 2, SCC-25, but not HSC-3 EVs, enhanced the cytotoxic activity of NK cells against both HSC-3 and SCC-25 cells (Figure 4H and 4K). Furthermore, NK cells from donor 3 induced more apoptosis in the HSC-3 cells when incubated with HSC-3 and SCC-25 EVs, while this ability against SCC-25 only appeared when incubated with SCC-25 EVs (Figure 4I and 4L).

We also noted an inhibition of the cytotoxic activity of CD8^+^ T and NK cells in three cases (Figure 4D, 4G, and 4J). In two of these cases, SCC-25 EVs resulted in this effect, while the third stemmed from HSC-3 EVs.

Finally, we found that EVs from less aggressive SCC-25 cells more effectively enhanced the cytotoxic activity of immune cells in 7 of 12 cases (Figure 4A, 4C, 4F, 4H, 4I, 4K, and 4L), while EVs from more aggressive HSC-3 cells induced an immune activity in only 4 of 12 cases (Figure 4B, 4C, 4F, and 4I).

### The effect of EVs from OTSCC cells on the innate immune system of zebrafish

We injected HSC-3 and SCC-25 EVs into the blood circulation of zebrafish larvae at 48 hours post-fertilization, and then stored the larvae for 24 hours before collecting RNA to analyze the expression of selected pro- and anti-inflammatory cytokines.

**Figure 5:**
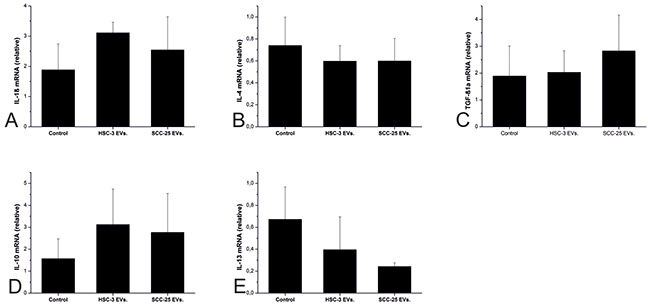
The effects of the OTSCC cell EVs on the pro- and anti-inflammatory cytokines in a zebrafish larvae model Zebrafish larvae were injected with PBS (control), HSC-3 EVs, and SCC-25 EVs and left for 24 hours. We measured the mRNA expression for IL-1β, **(A)** IL-4, **(B)** TGF-β1a, **(C)** IL-10, **(D)** and IL-13 **(E)** using q-PCR. Twenty five larvae were injected in each group and each experiment was repeated 3 times independently.

The levels of TNF-α and IFN-γ mRNA were undetectable across all groups (data not shown). IL-1β, IL-4, TGF-β1a, and IL-10 mRNA levels were unaffected by cancer cell EVs (Figure 5A–5D). However, SCC-25 steadily diminished the IL-13 mRNA level to one-third (Figure 5E).

## DISCUSSION

This study provides an *in vitro* 3D human TME matrix model in which immune cells were co-cultured with cancer cells on human uterus leiomyoma tumor discs or a soluble myoma matrix. These novel methods were designed to provide a more realistic simulation of the human TME than previously described *in vitro* models primarily using the mouse Engelbreth-Holm-Swarm tumor matrix, Matrigel, or rat-tail collagen [11, 12]. The study here shows the ability of immune cells to influence cancer cell proliferation and invasion in 3D models. It also highlights the importance of cancer cell EVs in enhancing the cytotoxic activity of immune cells and the expression of some anti-inflammatory cytokines using a zebrafish larvae model.

Studying the interaction between immune and tumor cells poses challenges since cancer cells in solid tumors are adherent, while most immune cells are non-adherent rendering their co-culture difficult. Furthermore, the co-culture environment is not representative of *in vivo* conditions when completed on plastic. Our *in vitro* model relies on human hypoxic myoma discs, which exhibit superior features in studying cancer cell invasion over classic organotypic models [11, 13]. In a myoma disc model, we seeded cancer cells on top of discs, whereas immune cells were added below to evaluate cancer cell chemotaxis towards immune cells. This effect was simultaneously mild and selective. In the majority of experiments, adding activated MNCs (with PMA and ionomycin) increased the number of CD45^+^cells in the myoma discs. However, we found substantial variation between samples. In fact, the attraction of CD8^+^ T cells appeared more distinct since their number increased significantly only in the HSC-3 myoma experiments. This indicates a selectivity among cancer cells to attract MNCs. However, CD8^+^ T cells were primarily found at the bottom and around the blood vessels of myoma discs. This suggests that MNCs use myoma disc blood vessels, as well as the inability of these cells to travel from the bottom towards superficial cancer cells. This may result from a weaker chemotaxis signal from the surface cancer cells compared to the chemokines produced by other bottom MNCs [14]. Alternatively, this may result from the loss of inflammatory vascular responses, including altering vascular permeability and oncotic pressure. These factors control the accumulation of fluids, molecules, and inflammatory cells and play crucial roles in the initial steps of chemotaxis and inflammation [15, 16]. Increase in the number of CD8^+^T cells was associated with the decrease in the cancer cells proliferation and their invasion area. Our results are in line with others who showed positive correlation between the number of the CD8^+^T cells and the survival time of the oral cancer patients [17, 18].

In addition, our results revealed the importance of stimulatory signals in controlling the type and fate of immune responses against cancer cells, since non-activated MNCs were unable to affect HSC-3 and SCC-25 cells for any of the features we studied. The most significant effect we observed occurred when MNCs were activated using PMA and ionomycin, while CD3 and CD28 antibodies showed a partial effect only on the proliferation of SCC-25 cells.

MNCs in general and lymphocytes in particular produce several soluble factors when subject to PMA and ionomycin treatment [19, 20]. Therefore, inhibiting cancer cell proliferation may result from soluble factors secreted from activated MNCs. Indeed, others found that soluble factors from embryonic and mesenchymal stem cells, for instance, can inhibit cancer cell proliferation [21–23]. The positive correlation between cancer cell proliferation and the invasion area indicates that a reduction in the proliferation area results from a decrease in cancer cell proliferation. Neither the invasion depth nor cancer cell migration were affected by activated MNCs. This may indicate an insignificant effect of activated MNCs on inhibiting cancer invasion and metastases formation [24]. Furthermore, cancer cell migration was only affected by PMA and ionomycin. A similar effect did not occur when these compounds were added to the media under myoma discs since they were not in direct contact with cancer cells. In both of our OTSCC cell lines, cell proliferation positively correlated with the invasion area, but not with the invasion depth, indicating that more cancer cells do not necessarily lead to a higher probability of invasion and metastases [24].

Because cancer cell EVs are known to significantly affect cells in TME [25], the second part of this study focused on cancer cell EVs on the immune system. We analyzed the effect of cancer cell EVs on macrophage polarization, CD8^+^ T and NK cell cytotoxicity, and the innate immune system *in vivo* using a zebrafish model. M2 (tumor-associated) macrophages, when present in TME, correspond with a poor prognosis in several cancers, including tongue cancer [4, 26, 27]. Cancer cells through the conditioned media can polarize primary macrophages and the human monocytic cell line (THP-1) towards M2 [28, 29]. We investigated whether this polarization effect could also be achieved using cancer cell EVs. While our pilot experiment using THP-1 cells incubated with HSC-3 EVs indicated a clear shift towards M2 (data not shown), we did not obtain similar results when we tested primary human macrophages. The difference between cancer cell–conditioned media and their EVs vis-à-vis affecting macrophage polarization may result from isolated EVs being too weak compared with the complete conditioned media, which is rich with cytokines and growth factors needed for macrophage polarization. These differences in the responses between THP-1 and primary macrophages agree with previous reports demonstrating that the THP-1 cell line is more responsive to stimulants compared with primary monocytes [30]. Therefore, THP-1 results may not accurately represent results of the primary cells.

Cancer and dendritic cell EVs can be used to activate the immune system against cancer through a mechanism similar to that in vaccines [31]. Here, we isolated EVs from more and less aggressive OTSCC cell lines (HSC-3 and SCC-25, respectively) and incubated them with adaptive immunity CD8^+^ T cells and innate immunity NK cells followed by immune cell co-culturing with OTSCC cell lines. Interestingly, the CD8^+^ T and NK cell responses to cancer cell EVs varied depending on several factors. The cytotoxic activity of the immune cells fluctuated depending on the donor immune cells, OTSCC cell line EVs, and against which OTSCC cells tested. Our results reflect the immune system fluctuations from person to person based on both genetic and epigenetic factors. This agrees with a review by Kunigelis et al. [31], who concluded that tumor EVs can exert contradictory effects (activating and inhibiting) on immune cells such as macrophages, CD4^+^ and CD8^+^ T cells, and dendritic cells. This presents a challenge to the use of EVs for clinical purposes.

Zebrafish increasingly serve as a model for studying cancer, particularly the crosstalk between cancer cells and the immune system [32, 33]. Like other non-mammalian model organisms, the zebrafish model is efficient, economical, and ethical. It also provides a fully developed vertebrate immune system closely resembling mammalian immunity given the similarities of the innate immune system of zebrafish to the mammalian system. In addition to that, all immune cell types are shared, including the neutrophils, macrophages, monocytes, dendritic cells, basophils, mast cells, and eosinophils [34–36]. The basic ethical principle guiding the model organism choice is to choose the one suitable for answering the research question and with the lowest degree of neurophysiological sensitivity. We chose the zebrafish larval model because it not only appears highly suitable, but also represents the most ethical model for this study. In order to understand the effect of cancer cell EVs on the innate immune system, we studied several pro- and anti-inflammatory cytokines. Cytokines represent major players in cancer progression, first through their direct effect on cancer cells, and second through their effect on TME, particularly immune cells [35]. Anti-inflammatory cytokines typically associate with a poor prognosis, primarily due to their inhibitory effect on the immune system [38, 39]. Our results indicated that HSC-3 and SCC-25 EVs were unable to alter pro-inflammatory cytokine expression. By contrast, cancer cell EVs decreased the level of IL-13, an anti-inflammatory cytokine. In fact, our results are not in line with a previous report that showed an increase in the level of IL-13 in the saliva of OSCC patients [38]. This indicates that not all elements of TME, including cancer cell EVs, induce the same direct effect on the innate immunity.

Taken together, our results along with previous reports support the need for a personalized approach to cancer treatment. This treatment should be based on testing the chemoradiation options available using a patient's cancer cells supplied with patient's serum, containing, for instance, EVs and cytokines. This test should consist of a 3D organotypic model instead of conventional 2D cell cultures on plastic. Compared to previous*in vitro* models, our 3D human TME-simulating models permit us to combine cancer cells with several cancer-associated cell types (such as immune cells) and soluble factors (including EVs) to better represent the complexity of solid cancers.

## MATERIALS AND METHODS

### Isolation of the human peripheral blood MNCs from the buffy coat of healthy donors

Human peripheral blood MNCs were isolated from a buffy coat provided by the Finnish Red Cross. A density gradient technique was followed to isolate MNCs using Ficoll–Paque PLUS (GE Healthcare, Piscataway, NJ, USA).

A MACS system (Miltenyi Biotec, Bergisch Gladbach, Germany) with a negative selection was used to isolate CD8^+^ T cells, CD14^+^ monocytes, and CD56^+^ NK cells from the peripheral blood MNCs of three healthy donors according to the manufacturer's protocol.

To check the purity of the isolated cells, different cell populations were stained using a panel of antibodies. This panel included anti-CD3-APC, anti-CD8-PerCP-Cy5.5, anti-CD14-FITC, and anti-CD56-PE (BD Biosciences, San Jose, CA, USA). Stained cells were acquired with FACS–Verse and analyzed using the BD FACSuite™ software.

CD3^+^ CD8^+^ T cells, CD3^−^ CD14^+^ monocytes, and CD3^−^ CD56^+^ NK cells formed 96%, 92%, and 92%, respectively, of the selected population (Supplementary Figure 3).

### Cell culture of human OTSCC cells

Two human OTSCC cell lines, HSC-3 (Japan Health Sciences Foundation, Japan) and SCC-25 (American Type Culture Collection, Manassas, VA, USA), were used in this study. HSC-3 cells were highly invasive, while SCC-25 cells were poorly invasive [40]. The cells were cultured in 75 cm^2^ flasks containing Dulbecco's modified Eagle's medium (DMEM)-12 (Gibco, Paisley, UK) supplied with 10% heat-inactivated fetal bovine serum (FBS), 100 U/ml penicillin, 100 μg/ml streptomycin, 250 ng/ml fungizone, and 50 μg/ml ascorbic acid.

### Co-culture of human peripheral blood MNCs with human OTSCC cells using organotypic myoma discs

In order to study the effect of peripheral blood MNCs on the proliferation and invasion of OTSCC cells and the ability of cancer cells to attract MNCs, we developed an *in vitro* model using human uterine myoma discs. The Ethics Committee of Oulu University Hospital approved our use of myoma tissue and all patients signed an informed consent form. We prepared uterine myoma discs and completed invasion experiments with some modifications as described previously [11]. Myoma discs do not have any live cells since they all die during the preparation process [41]. In brief, we placed myoma discs into Transwell® inserts (Corning Inc., Corning, NY, USA) and 50 × 10^4^ HSC-3 and SCC-25 cells were seeded on top of the discs. The cells were allowed to adhere overnight, and, on the next day, the discs were transferred onto a steel grid in a 12-well plate supplied with 1.2 ml culture media replaced every 3 to 4 days for 10 days. On the fourth day, 20 × 10^5^ MNCs were added under the myoma discs either alone (non-activated), with 2 μg/ml CD28 antibody (BD Bioscience) in a well coated with 5 μg/ml CD3 antibody (BD Bioscience, to activate CD3^+^ T cells) or with 5 ng/ml PMA (Sigma Aldrich, St. Louis, Mo, USA,) and 1 μM ionomycin (BD Bioscience, to activate all MNCs). For controls, cancer cells were cultured either with media alone or with media supplied with 5 ng/ml PMA and 1 μM ionomycin.

### Immunohistochemical staining of the myoma discs

Myoma discs were fixed in 10% formalin and embedded in paraffin blocks. All samples were cut into 6-μm-thick sections and stained using a fully automated Leica BOND-MAX staining robot (Leica Microsystems, Wetzlar, Germany) and a horseradish peroxidase–labeled dextran polymer method. As primary antibodies, we used monoclonal mouse anti-human pan-cytokeratin (0.7 μg/ml), Ki67 (0.8 μg/ml), CD45 (0.94 μg/ml), and CD8 (3 μg/ml; all from DAKO, Glostrup, Denmark).

### Scratch-wound cell migration assay

To test the effect of peripheral blood MNCs on OTSCC cell migration in a 3D cell culture, wells of a 96-well Imagelock plate (Essen Bioscience, Ann Arbor, MI, USA) were coated with 300 μg/ml Myogel, a matrix derived from human myoma tissue to replace Matrigel®, of mouse origin [12], and left overnight in the cell culture incubator. Excess Myogel was removed by suction and HSC-3 and SCC-25 cells were seeded at a density of 30 × 10^3^ cells/well and left to adhere overnight. We used the WoundMaker™ (Essen Bioscience) tool in order to achieve homogeneous scratch wounds. After making the wound, the media was replaced with 100 μl of fresh media and 80 × 10^3^ MNCs with or without stimulants (as in the organotypic model) were added. As a control, cancer cells were cultured with either media alone or with media supplied with 5 ng/ml PMA and 1 μM ionomycin. Wound confluence was monitored using the IncuCyte Live-Cell Imaging System (Essen Bioscience), and images were taken each hour.

### EV isolation and characterization

HSC-3 and SCC-25 cells were seeded at a density of 1.3 × 10^6^ per 75 cm^2^ flask and left to adhere overnight. The cell culture media was removed and the flasks were washed with phosphate buffered saline (PBS) before adding the serum-free DMEM-12 media. Cells were cultured for 48 hours and the media was then collected for EV isolation.

EVs were isolated using a two-step ultracentrifugation procedure. First, the conditioned media was centrifuged at 10 000 × g for 90 min at 4°C. The supernatant was then collected and centrifuged again at 100 000 × g for 90 min at 4°C. The second supernatant was discarded and the pellet containing EVs was re-suspended in 200 μl of PBS and kept at −80°C until further use.

Purified EV samples were analyzed by nanoparticle tracking analysis (NTA) using Nanosight model LM14 (Nanosight) equipped with a blue (404 nm, 70 mW) laser and scientific CMOS camera. The samples were diluted in Dulbecco's PBS and three 90-s videos were recorded using camera level 14. The data were analyzed using NTA software version 3.0 with the detection threshold optimized for each sample and the screen gain at 10 to track as many particles as possible with minimal background.

For immuno-electron microscope of the complete mount of EV samples, EVs were loaded on 200 mesh grids and fixed with 2% PFA. The samples were blocked with 0.5% BSA, in a 0.1-M NaPO4 buffer (pH 7.0) for 10 min and stained with anti-CD63 (Pelicluster, Sanquin, Amsterdam, The Netherlands) as well as 10-nm gold-conjugated anti-mouse secondary antibodies in 0.1% BSA in the same buffer for 30 to 60 min at room temperature. EVs were washed extensively before negative staining with 2% neutral uranyl acetate and embedded in a methyl cellulose–uranyl acetate mixture (1.8/0.4%). Samples were imaged using Tecnai 12 (FEI Company, Eindhoven, The Netherland) at 80 kV equipped with a Gatan Orius SC 1000B CCD camera (Gatan Inc. USA) using a 4008 × 2672 pixel image size and no binning.

The majority of EVs fell within the range of 50 to 100 nm and were CD63^+^ (Supplementary Figure 4).

### Monocyte differentiation and macrophage polarization

Human primary CD14^+^ monocytes were isolated from a buffy coat and tested for purity using flow cytometry. Monocytes were seeded at a density of 20 × 10^4^ in a 24-well plate. The culture medium was advance RPMI-1640 (Gibco) supplied with 10% FBS, 1% L-glutamine, 100 U/ml penicillin, 100 μg/ml streptomycin, and 250 ng/ml fungizone. The cells were allowed to adhere for 90 min, and unattached cells were then washed away with PBS and the wells were supplied with fresh media plus 100 ng/ml M-CSF (R&D Systems, Minneapolis, MN, USA). Monocytes were allowed to differentiate into macrophages for seven days, and images were taken each day using a Nikon DS-Fi2 camera (Nikon, Tokyo, Japan).

To study the effect of cancer cell EVs on macrophage polarization, differentiated macrophages were then subjected to EVs isolated from HSC-3 and SCC-25 cells for 24 hours. As a positive control, macrophages were differentiated to M1 macrophages using 10 ng/ml LPS (Sigma-Aldrich) and 20 ng/ml IFN-γ (Prospec, Rehovot, Israel), and to M2 macrophages using 20 ng/ml IL-4 and 20 ng/ml IL-13 (Prospec).

To check macrophage phenotypes using q-PCR, cells were lysed using an RLT buffer (Qiagen, Düsseldorf, Germany) and kept at −80°C until RNA isolation.

### Immune cell killing assay

The cytotoxic activity of CD8^+^ T and NK cells was tested against OTSCC cells using an immune cell killing assay. HSC-3 and SCC-25 cells were labeled with fluorescent dye using a CellTrace™ Far Red Cell Proliferation Kit (Invitrogen, Carlsbad, CA, USA), seeded at a density of 5000 cells in a 96-well plate and left to adhere overnight. CD8^+^ T and NK cells were cultured either with media alone or with media supplied with HSC-3 and SCC-25 EVs for 24 hours. As activators, CD8^+^ T cells were activated by 100 ng/ml CD3 antibody and 10 ng/ml IL-2 (eBioscience, San Diego, CA, USA), while for NK cells only we used 10 ng/ml IL-2. Next, 50 × 10^3^ NK and CD8^+^ T cells were added to the cancer cells (10:1 ratio) in the presence of 2.5-μM IncuCyte™ Caspase-3/7 Apoptosis Assay Reagent (Essen Bioscience). The plate was placed in the IncuCyte Live-Cell Imaging System for five days and images were taken every two hours.

### Microinjection of EVs into zebrafish larvae

Wild-type zebrafish of the AB strain were maintained at 28.5°C as described previously [42]. The larvae were grown at +28.5°C in an embryonic medium (5 mM NaCl, 0.17 mM KCl, 0.33 mM CaCl2, 0.33 mM MgSO4, and 10–15% Methylene Blue; Sigma-Aldrich). At 48 hours post-fertilization, zebrafish larvae were dechorionated, anaesthetized with 0.04% Tricaine, and 1 nl of HSC-3 or SCC-25 EVs or PBS was microinjected into the blood circulation valley (Duct of Cuvier). Larvae were transferred to a fresh embryonic medium and kept at +28.5°C for 24 hours and then collected for RNA isolation. Each group consisted of 25 larvae and each experiment was repeated 3 times independently.

### Quantitative real-time PCR

Two hundred nanograms of RNA were used for cDNA synthesis using an iScript cDNA synthesis kit (Bio-Rad, Hercules, CA, USA). For PCR, 10 μl iQ SYBR green, 7 μl water, and 1 μl of 250 nM primer solution were added to 2 μl of a cDNA sample. Glyceraldehyde 3-phosphate dehydrogenase (GAPDH) and ribosomal protein lateral stalk subunit P0 (RPLP0) were used as housekeeping genes. The primer sequences appear in Supplementary Table 5.

### Microscopic and histomorphometric analysis

Stained sections were studied using a Leica DM6000 B/M light microscope connected to a digital camera (DFC420; Leica Microsystems).

The invasion area, which represents the total surface area of the invaded cancer cells excluding the surface non-invading cells, and depth were calculated using ImageJ software (Wayne Rasband, National Institute of Mental Health, Bethesda, MD, USA) as described previously [11]. The number of CD45^+^ and CD8^+^ T cells was calculated from at least three selected fields (10 or 20X magnification), which showed the highest infiltration for each slide. The percentage of Ki67^+^ cells was calculated from at least three randomly selected fields of the non-invading cells (on the myoma surface).

### Statistical analysis

All experiments were repeated independently three to eight times, each in duplicate or triplicate. Values are given as means ± standard deviations. To determine the statistical significance, we performed one-way analysis of variance (ANOVA) followed by Tukey's post-hoc test. We set statistical significance to *P* < 0.05. Correlations between cell proliferation, the invasion area, and depth were determined using the Pearson's correlation test.

## SUPPLEMENTARY MATERIALS FIGURES, TABLE AND VIDEOS



## Abbreviations

EVs: extracellular vesicles
DMEM: Dulbecco's modified Eagle's medium
FBS: fetal bovine serum
GAPDH: glyceraldehyde 3-phosphate dehydrogenase
IFN-γ: interferon gamma
IL: interleukin
LPS: lipopolysaccharide
M-CSF: macrophage colony–stimulating factor
MNCs: mononuclear cells
NTA: nanoparticle tracking analysis
OTSCC: oral tongue squamous cell carcinoma
PBS: phosphate buffered saline
PMA: phorbol 12-myristate 13-acetate
RPLP0: ribosomal protein lateral stalk subunit P0
TME: tumor microenvironment
