# Supplementary material for: Crosstalk between tongue carcinoma cells, extracellular vesicles, and immune cells in in vitro and in vivo models
Source: Oncotarget. 2017 May 10;8(36):60123–34. doi: 10.18632/oncotarget.17768 (PMC5601126; doi:10.18632/oncotarget.17768)
Supplement: Supplementary file 1 [file oncotarget-08-60123-s001.pdf]

# Crosstalk between tongue carcinoma cells, extracellular vesicles, and immune cells in *in vitro* and *in vivo* models

## SUPPLEMENTARY MATERIALS

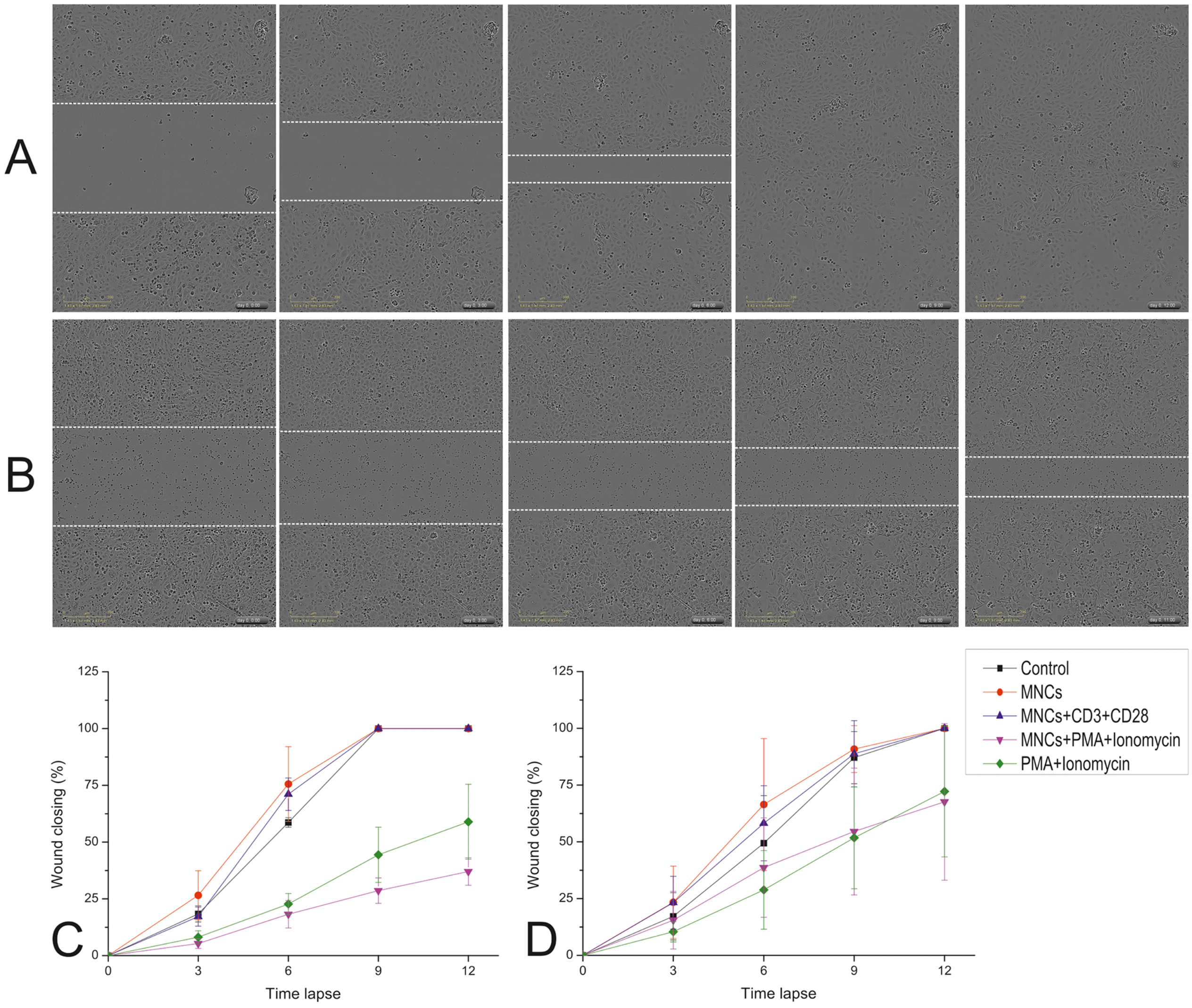


**Supplementary Figure 1: The effect of peripheral blood MNCs on the migration of OTSCC cells.** A scratch-wound cell migration assay was used to study the effect of peripheral blood MNCs on OTSCC cells migration. **(A)** Wound closing in the presence of HSC-3 cells alone. **(B)** Wound closing in the presence of HSC-3 cells with MNCs, PMA, and ionomycin. **(C)** Wound-closing percentage of HSC-3 cells in the presence and absence of MNCs. **(D)** Wound-closing percentage of SCC-25 cells in the presence and absence of MNCs.

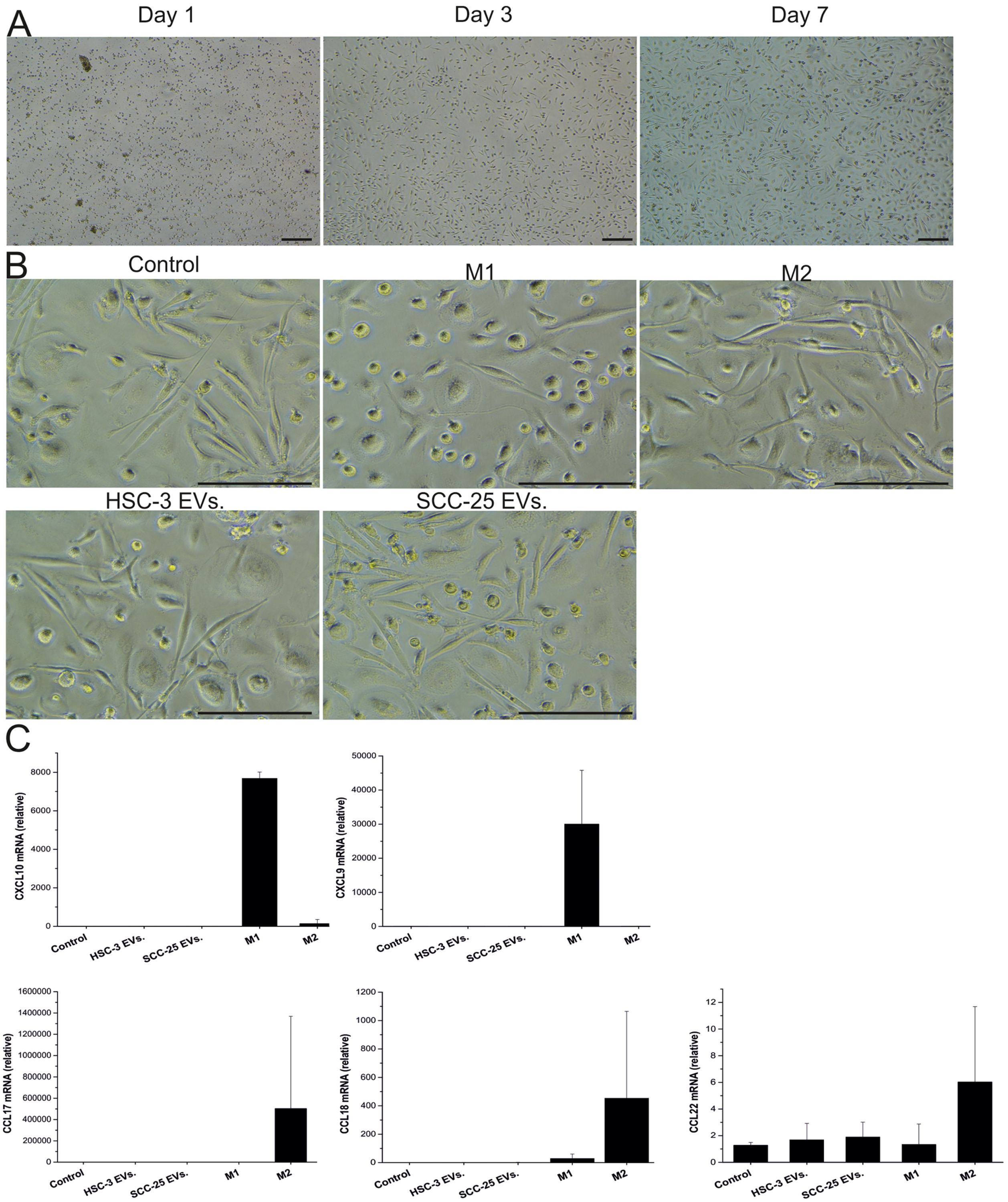


**Supplementary Figure 2: The effect of OTSSC cell EVs on human primary macrophage polarization.** $CD14^+$ monocytes were isolated from the buffy coats of three healthy donors and differentiated into macrophages for seven days in the presence of 100 ng/ml M-CSF **(A)**. Macrophages were incubated with media alone, LPS+IFN-γ (M1), IL-4+IL-13 (M2), HSC-3 EVs, or SCC-25 EVs **(B)**. The expression of M1 (CXCL9 and CXCL10) and M2 markers (CCL17, CCL18, and CCL22) was tested using q-PCR **(C)**. Scale bar = 200 µm.

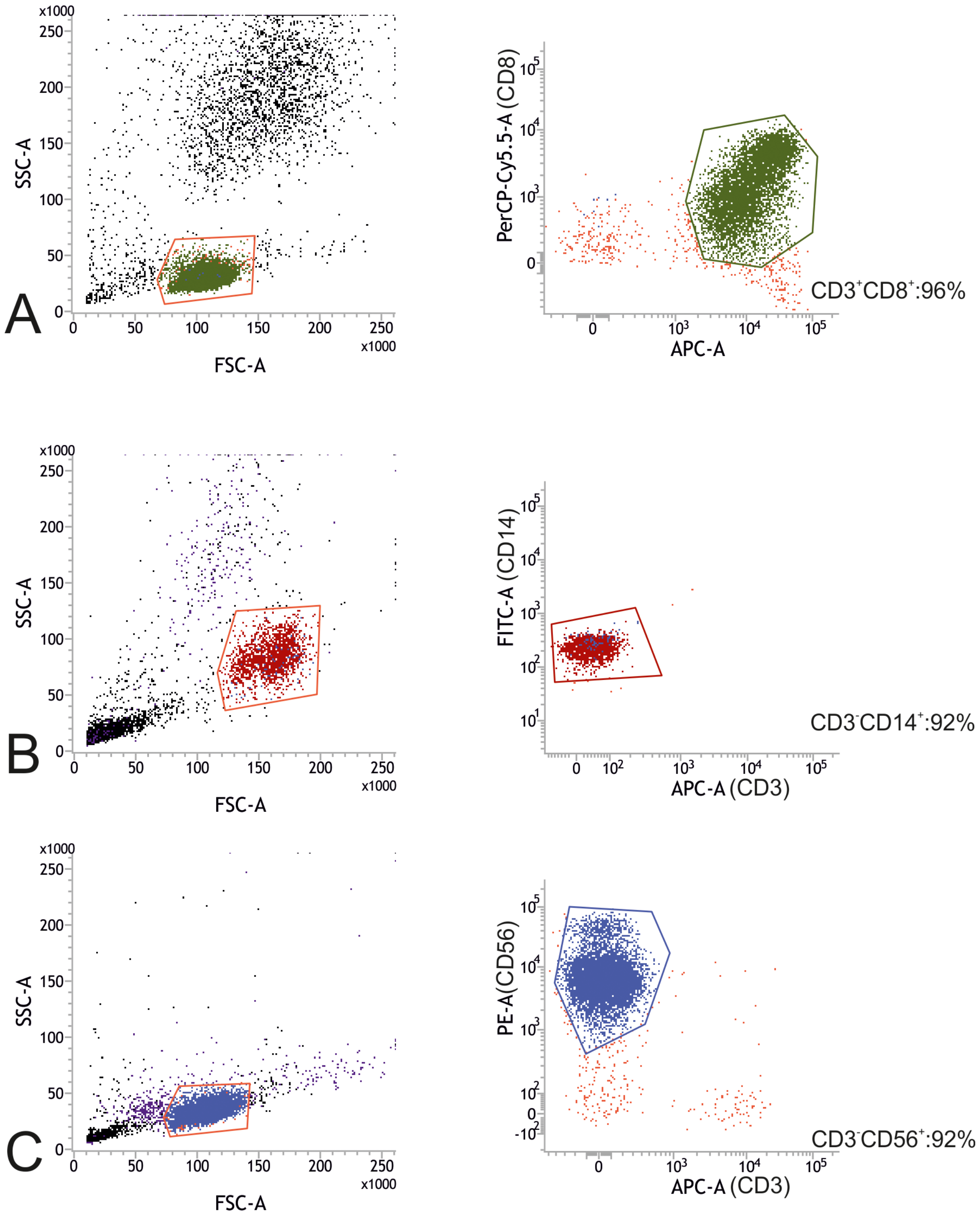


**Supplementary Figure 3: Testing cell purity using flow cytometry.** Isolated cells from a buffy coat were stained with anti-CD3-APC, anti-CD8-PerCP-Cy5.5, anti-CD14-FITC, and anti-CD56-PE, acquired using FACS-Verse and analyzed using BD FACSuite™ software. **(A)** $CD3^+$ $CD8^+$ T cells, **(B)** $CD3^-$ $CD14^+$ monocytes, and **(C)** $CD3^-$ $CD56^+$ NK.

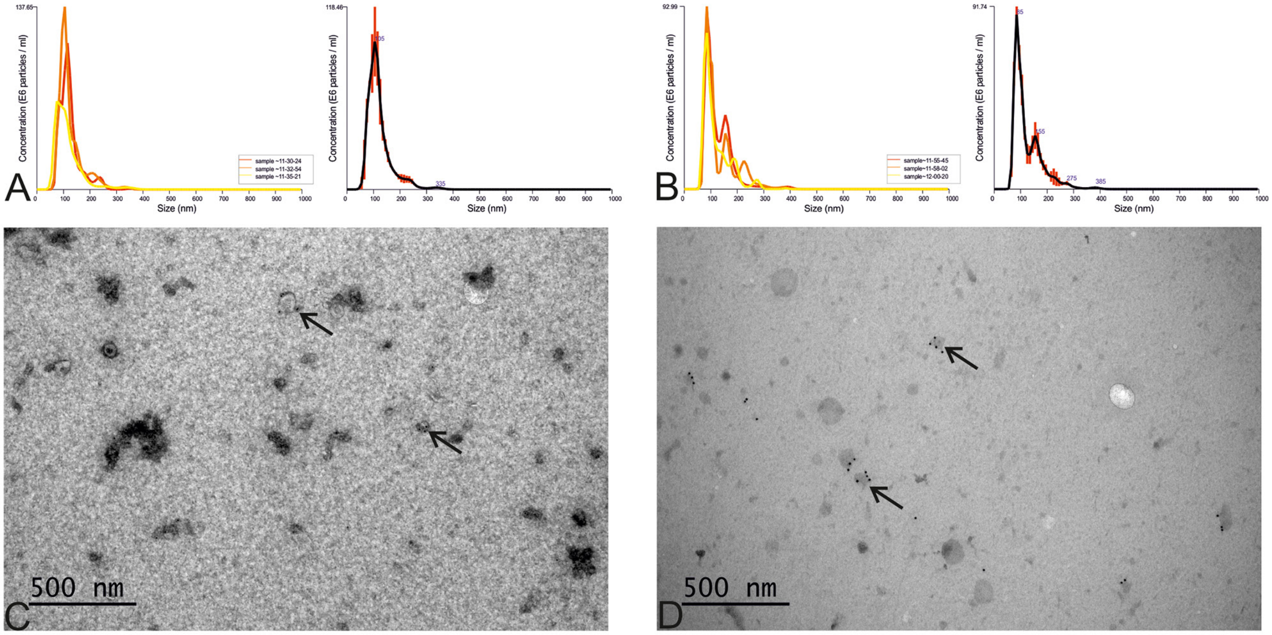


**Supplementary Figure 4: Characterization of OTSCC cell EVs using NTA and an electron microscope.** Purified EV samples were analyzed using NTA and stained with CD63, and then visualized by electron microscopy. The majority of EVs fell within the range of 50–100 nm in both HSC-3 **(A)** and SCC-25 **(B)**. Most EVs were positive for CD63 (arrow) in HSC-3 **(C)** and SCC-25 **(D)**.

| Species | Gene | Forward | Reverse |
|---|---|---|---|
| Human | CXCL9 | 5'-ACCCAGATTCAGCAGATGTGAAGGA-3' | 5'-GCCATCCTCCTTTGGAATGATAGCG-3' |
| Human | CXCL10 | 5'-GCAAGCCAATTTTGTCCACGTGTTG-3' | 5'-CAGCCTCTGTGTGGTCCATCCTT-3' |
| Human | CCL17 | 5'-GGACCTGCACACAGAGACTCC-3' | 5'-CCCTTGAAGTACTCCAGGCAGC-3' |
| Human | CCL18 | 5'-TGGCAGATTCCACAAAAGTTCA-3' | 5'-GGATGACACCTGGCTTGGG-3' |
| Human | CCL22 | 5'-GTGGCGCTTCAAGCAACTGA-3' | 5'-GGGGCAGACGGTAACGGAC-3' |
| Human | GAPDH | 5'-AAGGTCATCCCTGAGCTG-3' | 5'-TGCTGTAGCCAAATTCGTTG-3' |
| Human | RPLP0 | 5'-GGCGACCTGGAAGTCCAACT-3' | 5'-CCATCAGCACCACAGCCTTC-3' |
| Zebrafish | IL-1β | 5'-GTTCAGATCCGCTTGCAATGAGC-3' | 5'-AAGTCCACATCTCCAGCCTGATG-3' |
| Zebrafish | IL-4 | 5'-GCAGGAATGGCTTTGAAGGG-3' | 5'-GCAGTTTCCAGTCCCGGTAT-3' |
| Zebrafish | IL-10 | 5'-TTGGAGACCATTCTGCCAACAGC-3' | 5'-TGCATTTCACCATATCCCGCTTG-3' |
| Zebrafish | IL-13 | 5'-GGAAGCTGTGTTAGTCAATCC-3' | 5'-GCCTGACAGAAATAATCATGC-3' |
| Zebrafish | IFN-λ 1-2 | 5'-GGGCGATCAAGGAAAACGACCC-3' | 5'-TAGCCTGCCGTCTCTTGCGT-3' |
| Zebrafish | TGF-1βa | 5'-AAGTTCGTCTTCCAGCAAGCTC-3' | 5'-TACTGCGCTTCATCTCGGACAC-3' |
| Zebrafish | TNF-α | 5'-GGGCAATCAACAAGATGGAAG-3' | 5'-GCAGCTGATGTGCAAAGACAC-3' |
| Zebrafish | GAPDH | 5'-AGTGTCAGGACGAACAGAGGCT-3' | 5'-GCCAATGCGACCGAATCCGTTA-3' |

**Supplementary Table 5: Sequences of the human and zebrafish primers used in this study.**

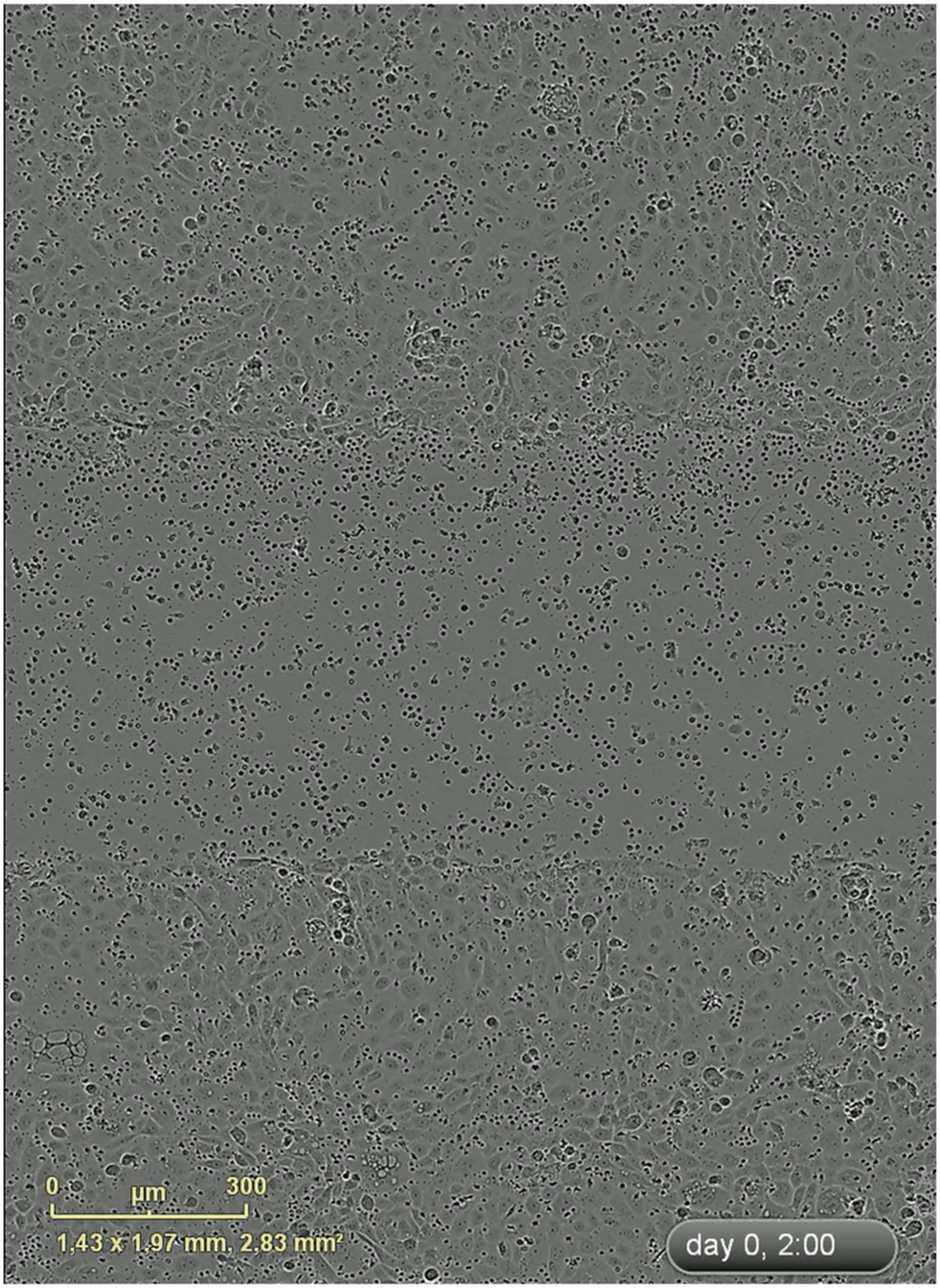


**Supplementary Video 1: Scratch-wound cell migration assay of the HSC-3 cells in the presence of MNCs.** Videos were taken using the IncuCyte Zoom Live-Cell Imaging System.

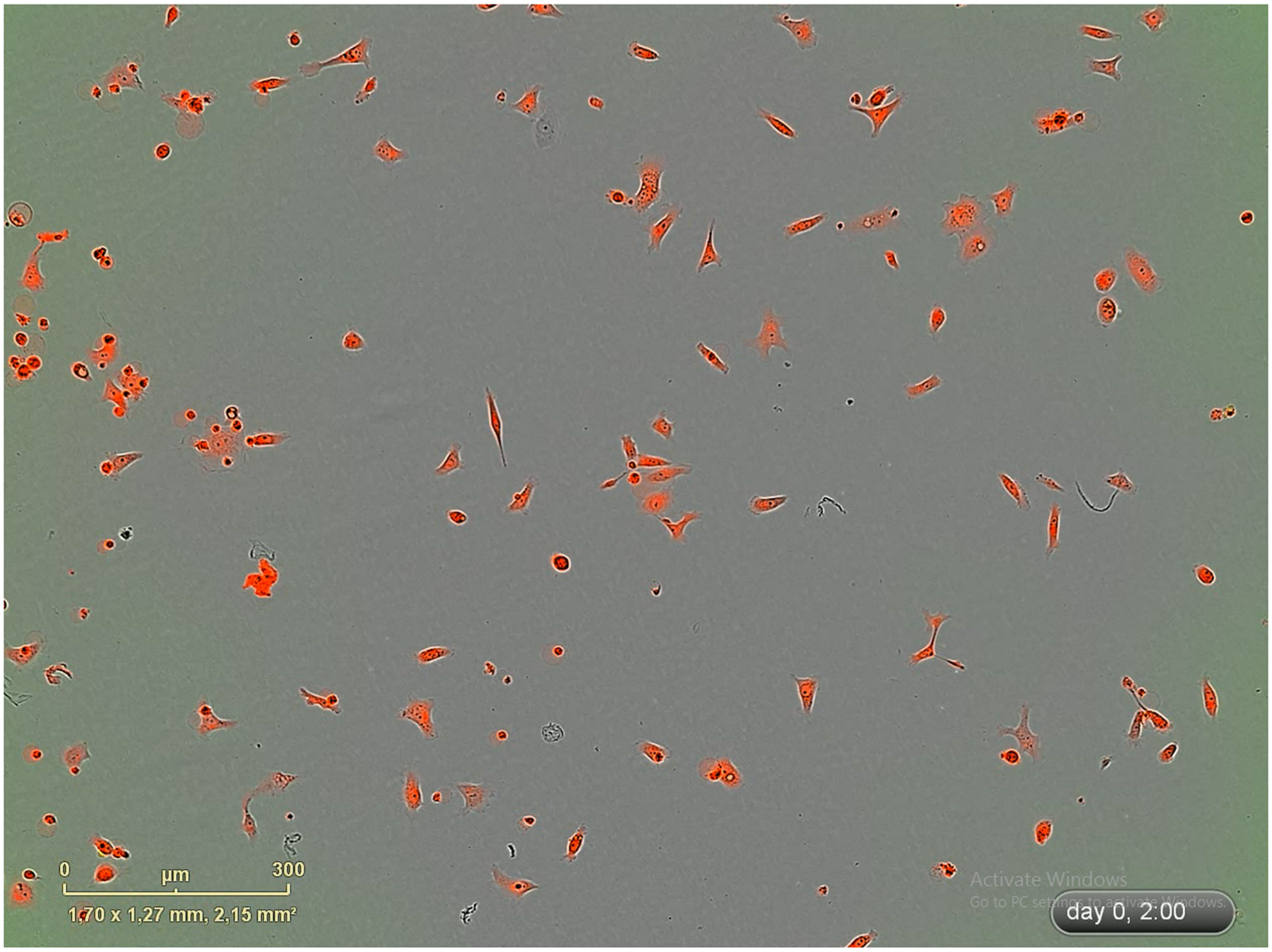


**Supplementary Video 2: Immune killing assay.** HSC-3 cells (red) were labeled with CellTrace™ Far Red and incubated in a 96-well plate for 5 days in the presence of the IncuCyte™ Caspase-3/7 Apoptosis Assay Reagent. Apoptotic cells appear in yellow in the video. Videos were taken using the IncuCyte Zoom Live-Cell Imaging System.

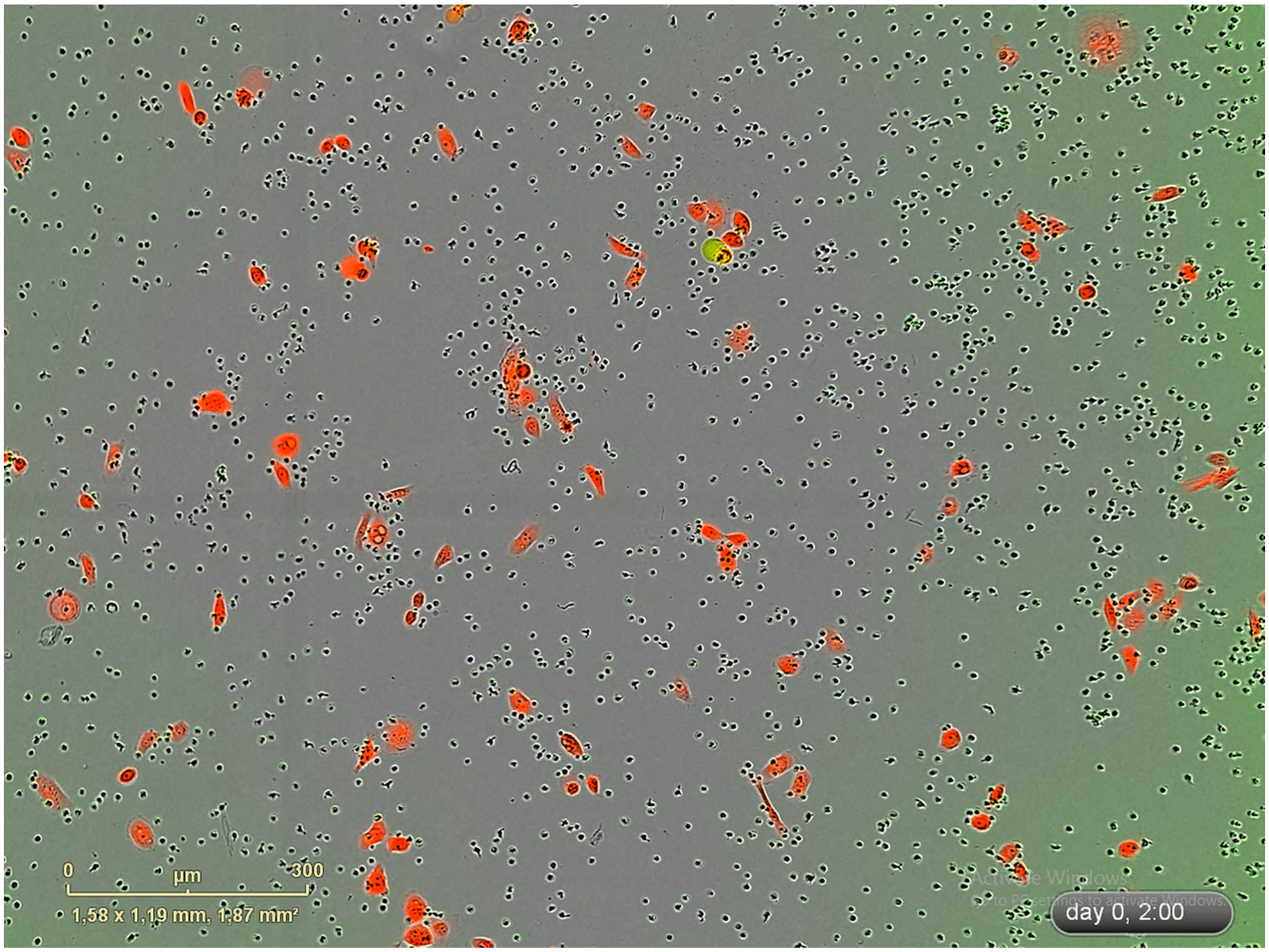


**Supplementary Video 3: Immune killing assay.** HSC-3 cells (red) were labeled with CellTrace™ Far Red and incubated with NK cells (black) in a 96-well plate for 5 days in the presence of the IncuCyte™ Caspase-3/7 Apoptosis Assay Reagent. Apoptotic cells appear in yellow in the video. Videos were taken using the IncuCyte Live-Cell Imaging System.
